# Together But Different: The Subgenomes of the Bimodal *Eleutherine* Karyotypes Are Differentially Organized

**DOI:** 10.3389/fpls.2019.01170

**Published:** 2019-10-07

**Authors:** Mariana Báez, Magdalena Vaio, Steven Dreissig, Veit Schubert, Andreas Houben, Andrea Pedrosa-Harand

**Affiliations:** ^1^Laboratory of Plant Cytogenetics and Evolution, Department of Botany, Federal University of Pernambuco, Recife, Brazil; ^2^Laboratory of Genetics, Department of Plant Biology, College of Agronomy, University of the Republic, Montevideo, Uruguay; ^3^Department of Breeding Research, Leibniz Institute of Plant Genetics and Crop Plant Research (IPK), Gatersleben, Germany

**Keywords:** retrotransposons, satellite DNA, repetitive sequences accumulation, DNA replication, histone modification, inversion

## Abstract

Bimodal karyotypes are characterized by the presence of two sets of chromosomes of contrasting size. *Eleutherine bulbosa* (2*n* = 12) presents a bimodal karyotype with a large chromosome pair, which has a pericentric inversion in permanent heterozygosity with suppressed recombination, and five pairs of three to four times smaller chromosomes. Aiming to understand whether high copy number sequence composition differs between both chromosome sets, we investigated the repetitive DNA fraction of *E. bulbosa* and compared it to the chromosomal organization of the related *Eleutherine latifolia* species, not containing the pericentric inversion. We also compared the repetitive sequence proportions between the heteromorphic large chromosomes of *E. bulbosa* and between *E. bulbosa* and *E. latifolia* to understand the influence of the chromosome inversion on the dynamics of repetitive sequences. The most abundant repetitive families of the genome showed a similar chromosomal distribution in both homologs of the large pair and in both species, apparently not influenced by the species-specific inversions. The repeat families Ebusat1 and Ebusat4 are localized interstitially only on the large chromosome pair, while Ebusat2 is located in the centromeric region of all chromosomes. The four most abundant retrotransposon lineages are accumulated in the large chromosome pair. Replication timing and distribution of epigenetic and transcriptional marks differ between large and small chromosomes. The differential distribution of retroelements appears to be related to the bimodal condition and is not influenced by the nonrecombining chromosome inversions in these species. Thus, the large and small chromosome subgenomes of the bimodal *Eleutherine* karyotype are differentially organized and probably evolved by repetitive sequences accumulation on the large chromosome set.

## Introduction

Bimodal karyotypes are characterized by the presence of two sets of chromosomes of contrasting size. The origin of bimodal karyotypes is usually associated with one of the following processes: (i) chromosomal rearrangements involving fusion–fission events generate large chromosomes as fusion products of small chromosomes, or small chromosomes result from the fission of large chromosomes (Burt, 2002; Schubert and Lysak, 2011; Yin et al., 2014). (ii) The combination of different parental species (allopolyploidization) may combine species with different chromosome sizes (McKain et al., 2012; Shirakawa et al., 2012). (iii) The differential accumulation of repetitive sequences may increase the size of a subset of chromosomes (de la Herrán et al., 2001). Bimodal karyotypes are common within several animals groups, such as birds, reptiles, and amphibians (Stock and Mengden, 1975; Masabanda et al., 2004; Noronha et al., 2016). Also, many plant genera, such as *Agave*, *Yucca*, *Hosta* (Akemine, 1935; Watkins, 1936; Palomino et al., 2012), *Aloe* (Brandham and Doherty, 1998; Fentaw et al., 2013), and *Hypochaeris* (Fiorin et al., 2013), show bimodal karyotypes.

In animal bimodal karyotypes, gene content, the abundance of heterochromatic repetitive sequences, and the replication behavior differ between both chromosome sets (McQueen et al., 1998; Smith et al., 2000). For instance, chicken microchromosomes are early replicating, harbor twice as many genes as macrochromosomes, and are associated with an increased gene transcriptional activity (McQueen et al., 1998). In contrast, in most bimodal plant groups, the chromosome organization is largely unknown. The bimodal karyotypes of some Orchidaceae species contain large chromosomes with a higher proportion of C-banding–positive heterochromatin (D’Emerico et al., 1999). In *Ornithogalum longibracteatum* (Hyacinthaceae), one satellite DNA sequence (satDNA) is the major constituent of the heterochromatin of the large chromosomes (Pedrosa et al., 2001). A specific satDNA, found in *Muscaricomosum* (Hyacinthaceae), is related to the heterochromatic bands of the large chromosomes, and it has been suggested to cause the increase of asymmetry of the karyotypes within this genus (de la Herrán et al., 2001). Independent of composition and origin, in both animal and plant bimodal species, it was suggested that the maintenance of these chromosome size differences could be related to the genome structure and function (Coullin et al., 2005; Vosa, 2005; Griffin et al., 2015).

*Eleutherine* (Iridaceae) is a neotropical genus of the subfamily Iridoideae and comprises two species, both with bimodal karyotypes (Goldblatt and Snow, 1991). *Eleutherine bulbosa* (2*n* = 12) has one chromosome pair (chromosome I), which is three to four times larger than the other pairs. The large chromosome pair is heteromorphic due to an asymmetric pericentric inversion in heterozygosity, encompassing about 70% of the chromosome and resulting in one acrocentric and one metacentric homolog (Guerra, 1988). This pair contains two DAPI-positive heterochromatic bands. They are located interstitially in the long arm of the acrocentric and terminally in the short arm of the metacentric homolog. CMA-positive bands are located in the pericentromeric region of both homologs. The presence of rDNA sites is limited to chromosome pair I. While the 35S rDNA sites are located inside of the chromosomal inversion, the 5S rDNA sites are duplicated in the terminal region of the long arm of both chromosomes, outside of the inversion (Feitoza and Guerra, 2011). The second species of the genus, *Eleutherine latifolia*, has also a 2*n =* 12 bimodal karyotype with a pair of large acrocentric chromosomes, but without an inversion (Goldblatt and Snow, 1991).

All small chromosomes of *E. bulbosa* are enriched in euchromatin marks, like acetylated histone H4K5 and dimethylated H3K4. In contrast, the large chromosome pair is 5-mC hypermethylated (Feitoza and Guerra, 2011), showing a chromatin differentiation between both chromosome sets. Meiotic analysis showed that the inverted region of the large chromosome pair was devoid of recombination, with chiasmata observed only outside the inversion loop (Guerra, 1991). All analyzed individuals and populations of *E. bulbosa* were heterozygous, and this heterozygosity is supposed to be fixed preferentially by asexual reproduction (Guerra, 1988; Guerra, 1991).

The process of recombination is linked to the evolution of repetitive sequences, as observed for satellite DNA homogenization *via* gene conversion (Feliner and Rosselló, 2012). Furthermore, unequal recombination between homologous chromatids or illegitimate recombination was proposed as powerful mechanisms for removing repetitive sequences (Tenaillon et al., 2010) and decreasing genome size (Renny-Byfield et al., 2011). Thus, chromosomal regions devoid of recombination, such as inverted regions, could tend to accumulate different types of repetitive sequences, which may evolve differentially from the rest of the genome. Within this context, the 5-mC hypermethylation of *E. bulbosa* chromosome pair I, which contrasts to the small chromosome pairs, and the lack of recombination between the homologs of chromosome pair I in a large segment led to the following questions. Does the distribution of repetitive sequences, epigenetics histone marks and timing of DNA replication differ between large and small chromosomes within a bimodal karyotype? Does the repetitive composition differ in the nonrecombining inverted region between the homologs of chromosome pair I? Is the distribution of repeats conserved between large and small chromosomes within the *Eleutherine* species?

Therefore, we describe the repeat composition and chromosome organization of *E. bulbosa*. The findings were compared to the sister species *E. latifolia*, also showing a bimodal karyotype but lacking the large chromosome inversion.

## Materials and Methods

### Materials

Plants of *E. bulbosa* were collected in Aracuara, Bahia State, Brazil (voucher number UFP 82763), and cultivated in the experimental garden of the Laboratory of Plant Cytogenetic and Evolution from the Federal University of Pernambuco, Recife, Brazil. Seeds of *E. latifolia* were kindly provided by Dr Guadalupe Munguía Linno from Guadalajara University, Mexico. Seeds were germinated in a wet chamber (3–4 months), the seedlings were transferred into soil and cultivated in a germination room at 24°C.

### Genome Size Estimation

Samples were prepared from 40 to 50 mg of young leaves of *E. bulbosa* (Miller) Urban or *E. latifolia* (Standl and L. O. Williams) Ravenna in 1 mL of LB nuclear isolation buffer and filtered through a 30 µm nylon filter (Doležel et al., 2007). *Solanum lycopersicum* L. (2C = 1.96 pg) served as standard. Nuclei were stained with propidium iodide (50 µg mL^−1^), and RNase (50 mg mL^−1^) was added to prevent staining of double-stranded RNA. The nuclear DNA content was determined with a Partec CyFlow SL (Partec) flow cytometer, and results were analyzed with Flomax program. For genome size estimations, three replicates were analyzed, and the nuclear DNA content for each species was calculated according to the formula:

2C nuclear DNA content of the sample (pg)=sample G0/G1reference standard G0/G1 ×2C nuclear DNA content of the reference standard

### Extraction of Genomic DNA and DNA Isolation From Microdissected Chromosomes

Total genomic DNA was extracted from young leaves of one individual of *E. bulbosa* using the DNAeasy Plant Mini Kit (Qiagen) according to manufacturer’s instruction.

For microdissection of the two large homologous chromosomes, root tips were collected from bulbs, pretreated in 8-hydroxyquinoline at 10°C for 24 h and fixed in 2% formaldehyde for 15 min under vacuum. Fixed root tips were chopped in a nuclei isolation buffer (Doležel et al., 2007) and filtered through a 30-µm nylon membrane. The cell solution was centrifuged onto a microscopic slide at 2,000 revolutions/min (rpm) for 10 min (Shandon, CytoSpin3). Slides, mounted in 4′, 6-diamidino-2-phenylindole (DAPI), were hit with a metal needle to physically separate chromosomes from broken cells. Ten chromosomes of each large chromosome homolog were isolated by microdissection with a glass needle, using a Zeiss Axio Zoom.V16 microscope coupled to an AxioCam 289 MRc5 digital camera (Zeiss) and the aureka^®^ microsampling platform (aura-optik). The chromosomal DNA was amplified by multiple displacement amplification according to the protocol described in Dreissig et al. (2015) with minor modifications. Briefly, chromosomes were collected in 0.5 µL H_2_O and 1 µL sample buffer (GE Healthcare, Genomiphi V2) and then were incubated in alkaline lysis buffer (Gole et al., 2013) and 0.1 µg/µL of proteinase K (Sigma) at 37°C for 1 h, followed by heat inactivation at 65°C for 10 min. After incubation, 0.5 µL of neutralizing buffer (Gole et al., 2013) was added, and the samples were left on ice, while a master mix was prepared (3.5 µL of sample buffer, 4.5 µL reaction buffer and 0.5 µL of enzyme mix; Genomiphi V2; GE Healthcare). The samples were incubated at 30°C for 8 h followed by heat inactivation at 65°C for 10 min and then cooled down to 4°C and kept at −20°C. A primer pair specific for CL29 of *E. bulbosa*, an LTR Ty3/Gypsy-Tat repeat (Online Resource 1), was used to check whether the generation of chromosome-derived DNA was successful.

### Next-Generation Sequencing and Sequences Analysis

Genomic DNA and chromosome microdissection-derived DNA were used for paired-end, 100-bp reads, and single-end, 250-bp reads, Illumina sequencing, respectively (Genbank Bioproject PRJNA549830). The repetitive fraction analysis was performed with 400 Mbp of reads of the genomic DNA (0.32× genome coverage) and 2,160 Mbp for each homolog of the large chromosome pair (∼8× genome coverage). Sequenced reads were analyzed with the similarity-based read clustering method, implemented in the RepeatExplorer pipeline (Novak et al., 2013). Reads were filtered by quality with the default sets (quality cutoff value = 10, within a 95% of the bases in the sequence), and genomic paired-end reads were joined with the interlaced tool. For single-end reads, datasets from both chromosome types were assigned a unique identifier and joined into a single dataset with the concatenate tool. For both datasets (genomic and chromosomes), clustering was performed with a minimum overlap of 55% and a similarity of 90%. For sequences of microdissected chromosomes, three independent analyses were performed, using a different dataset of reads of the same sequencing, to confirm the proportions of each cluster on the two different homolog chromosomes. Repeat annotation and classification were performed for those clusters with an abundance >0.01%. For basic repeat classification, protein domains were identified using the tool “Find RT Domains” in RepeatExplorer (Novak et al., 2013). Searches for sequence similarity, using different databases (GenBank and TIGR), were performed, and graph layouts of individual clusters were examined using the SeqGrapheR program (Novak et al., 2013). Satellite DNAs were identified based on the graph layout and further examined using DOTTER (Sonnhammer and Durbin, 1995).

### Amplification, Cloning, and Sequencing

The seven most abundant repeats of the total genome, three satellite DNAs (satDNA: Ebusat1, Ebusat2, Ebusat3) and four LTR-retrotransposons (LTR-RT) (Ty1/Copia-Maximus and -Tork and Ty3/Gypsy-Tat and -Chromovirus), were polymerase chain reaction (PCR) amplified. In addition, one satDNA (Ebusat4) from microdissected acrocentric chromosome DNA was also PCR amplified. For satellite DNAs, primers were designed, facing outward of the repeat unit, for the consensus sequences and from the region where most of the reads were conserved. LTR-RT–specific primers were designed to amplify the Integrase domain, commonly used in chromosome analyses with repetitive sequences, for being suggested as the most conserved domain within the domains of the retrotransposons (**Table S1**). The conserved region of the integrase domain was identified using the SeqGrapheR program (Novak et al., 2013). Forty nanograms of genomic DNA was used for all PCR reactions with 1× PCR buffer (Invitrogen), 2 mM MgCl_2_, 0.1 mM of each dNTP, 0.4 µM each primer, 0.025 U *Taq* polymerase (Platinum *Taq* DNA polymerase; Invitrogen), and water. Polymerase chain reaction conditions were as follows 94°C 3 min, 30× (94°C 1 min, 55°C 1 min, 72°C 1 min), and 72°C 10 min. Polymerase chain reaction fragments were purified from a 1% agarose gel using the AxyPrep DNA gel extraction kit (Axygen Biosciences) and cloned with the pGEM^®^-T Vector cloning system (Promega) using JM109 *Escherichia coli* high-efficiency competent cells (Promega), following manufacturer’s instructions. One positive clone of each repetitive element was sequenced with a 3500 Genetic Analyzer Sanger sequencing platform at the Biosciences Center of the Federal University of Pernambuco for confirming its identity. Sequences were deposited in the GenBank database as MK228130-MK228135.

### Chromosome Preparation and Fluorescence *In Situ* Hybridization

Cloned satellite DNAs, rDNAs, and retrotransposons were labeled with either Cy3-dUTP, Cy5-dUTP, or **digoxigenin-11-dUTP** by nick translation using a nick translation mix (Roche, Brazil) or with DNase I (0.002 U) and DNA polymerase (4 U) enzymes following Kato et al. (2004). 35S rDNA sites were detected with the pTa71 clone from *Triticum aestivum* (Gerlach and Bedbrook, 1979). Clone D2 from *Lotus japonicus* (Pedrosa et al., 2002) was used to detect the 5S rDNA.

Chromosomes were prepared from root tips collected from bulbs, pretreated in 0.02 M 8-hydroxyquinoline at 10°C for 24 h and fixed in ethanol: acetic acid (3:1 v/v) for 2 to 24 h at room temperature and stored at −20°C. Fixed root tips were digested with 2% cellulase-20% pectinase for 90 min at 37°C, and squashed in a drop of 45% acetic acid. Fluorescent *in situ* hybridization was performed as described by Pedrosa et al. (2002). The hybridization mix contained 50% (v/v) formamide, 10% (w/v) dextran sulfate, 2× SSC, and 5 ng/µL of each probe. Slides were denatured at 75°C for 5 min, and the final stringency of hybridization was 76%.

Images were captured using a Leica DM5500 B microscope with a Leica DFC345 FX coupled camera and the LAS AF software. Images were edited with Adobe Photoshop CS5.

### Immunodetection of Histone Modifications and Active RNA Polymerase II

Antibodies for three different histone modifications were used: one euchromatic mark, rabbit anti-histone H3K4me3 (Abcam1012, diluted 1:300), and two pericentromeric chromatin marks: mouse anti-H3S10ph (Abcam 14955, diluted 1:2,000) and rabbit anti-H2AT120ph (Demidov et al., 2014, diluted 1:500). The latter antibody was developed for the same peptide as described in Dong and Han (2012). A mark for transcriptional activity was also applied: rat anti-RNAPIISer2ph (Millipore 04-1571, diluted 1:100). For immunostaining, root tips were pretreated with 2 mM 8-hydroxyquinoline for 24 h at 10°C. For RNAPIISer2 detection, nuclei were isolated from leaves. Both were fixed in freshly prepared 4% paraformaldehyde (dissolved in 1× PBS) for 30 min on ice and then washed three times for 15 min in 1× PBS on ice. Fixed root tips and leaves were chopped in a nuclei isolation buffer (Doležel et al., 2007) and filtered through a 30-µm nylon membrane. The cell suspension was used to prepare slides by centrifugation onto a microscopic slide at 2,000 rpm for 3 min (Shandon, CytoSpin3). Slides were incubated in 3% bovine serum albumin (BSA) for 30 min at 37°C. Primary antibodies, diluted in 1% BSA, were incubated overnight at 4°C and detected with Alexa 488–conjugated anti-rabbit (Dianova 711-545-152, diluted 1:200), Alexa 488–conjugated anti-mouse (Molecular probes A11001, diluted 1:200), Alexa 488–conjugated anti-rat (Dianova 112-545-167, diluted 1:200), or goat Cy3-conjugated anti-mouse (Dianova 115-165-062, diluted 1:300) antibodies in 1% BSA and incubated for 1 h at 37°C.

After immunostaining with H2AThr120ph and RNAPIISer2ph, fluorescence *in situ* hybridization (FISH) was performed subsequently to analyze the colocalization with the Ebusat2 satellite and the Ty3/Gypsy-Tat LTR-retrotransposon, respectively. Therefore, the slides were washed twice in 1× PBS, fixed in ethanol:acetic acid (3:1 v/v) for at least 24 h at room temperature in the dark, dehydrated, and prehybridized in 15 µL of DS20 (50% formamide, 10% dextran sulfate, 2× SSC) overnight at 37°C. Slides were washed in 2× SSC, dehydrated, and denatured in 0.2 N NaOH in 70% ethanol for 10 min at room temperature. Afterward, additional dehydration was performed, and the slides were hybridized with 50 ng of the probe in DS20 overnight at 37°C.

Images for histone modifications were captured using an epifluorescence microscope BX61 (Olympus) equipped with a cooled CCD camera (Orca ER, Hamamatsu). To achieve super-resolution for RNAPIISer2ph imaging, spatial structured illumination microscopy (3D-SIM) was applied using a 63/1.4NA Oil Plan-Apochromat objective of an Elyra PS.1 microscope system and the software ZEN (Carl Zeiss GmbH, Germany) (Weisshart et al., 2016). For histone modification marks, the fluorescence intensity was estimated along the chromosomes using the ImageJ software (Abràmoff et al., 2004). Intensity measurements were done at 10 or eight consecutive circles of ∼50 to 60 pixels each, along with both homologs of the large chromosomes pair and one small chromosome pair, respectively. Five metaphases per mark were measured, and a mean of the measurements of each position along the chromosomes was calculated. We defined a ratio between the intensity of DAPI and the histone modification fluorescence along the chromosomes.

### DNA Replication Analysis

DNA replication analysis was performed with the EdU kit (BCK-EdU 594-1, baseclick GmbH, Germany) following the manufacturer’s protocol. Root tips were collected and incubated in a humid chamber with a filter paper embedded in an EdU solution for 3 h at room temperature for the incorporation of the dNTP analog. After incorporation, root tips recovered in water for 30 min, were pretreated in 8-hydroxyquinoline for 24 h at 10°C, and were fixed in ethanol:acetic acid (3:1 v/v). The cell walls were digested by treating the root tips with an enzyme mix of 0.7% cellulase R10 (Duchefa C8001), 1% pectolyase (Sigma P3026), and 1% cytohelicase (Sigma C8274) for 90 min at 37°C. The squashing of chromosomes was performed in a drop of 45% acetic acid. Then, the slides were immersed into liquid nitrogen to remove the coverslips. The slides were incubated with 3% BSA for 20 min at room temperature and then with the detection mixture for 30 min at room temperature in the dark. Sequential FISH with the Ebusat 1 and Ty3/Gypsy-Tat LTR-RT probes was performed as described above. Images were captured as described above.

## Results

### Bimodal Karyotype of *E. bulbosa* Is Characterized by a Differential Chromosome-Type Specific Repeat Distribution

To characterize the repetitive DNA fraction of *E. bulbosa* (1C = 1.25 Gbp), its genome was sequenced at 0.32× genome coverage. Reads, comprising in total 400 Mbp, were grouped into 118,468 clusters containing from 2 to 90,876 reads. Clusters included 49.7% of all reads, with the major 257 clusters representing at least 0.01% of the genome each. The analysis revealed three major satellite DNA families (satDNAs), 11 transposable element families (LTR-retrotransposons and LINE), DNA transposons and ribosomal DNA sequences (**Table 1**). The largest clusters were identified as satDNAs: CL1 (Ebusat1) representing 4.99% of the genome with a complex repeat unit with variable length; CL2 (Ebusat2) representing 2.16% of the genome with a 261-bp repeat unit; and CL3 (Ebusat3) representing 1.4% of the genome. The LTR-like retrotransposons constituted approximately 28% of the genome, with the Ty3/gypsy superfamily exceeding 2.15-fold the genome proportion of the Ty1/copia superfamily. Within the former, Tat and Chromovirus were the only highly abundant lineages. Within Ty1/copia retrotransposons, eight lineages were identified, with Maximus and Tork being the most abundant.

**Table 1:** Proportion (%) of repetitive elements present in the total genome of *E. bulbosa*.

Repetitive element		Total genome (%)
Satellite	Ebusat 1	4.99
	Ebusat 2	2.16
	Ebusat 3	1.40
	Ebusat 4	—*
LTR-Ty3/Gypsy	Tat	14.26
	Chromovirus	1.76
LTR-Ty1/Copia	Maximus	1.78
	Tork	1.46
	TAR	1.23
	AleII	1.19
	Angela	0.98
	Ivana/Oryco	0.31
	Bianca	0.48
	AleI.Retrofit	0.031
Unclassified LTR		6.39
LINE		0.19
DNA Transposons		2.08
rDNA		0.53
Microsatellite		0.33
Unclassified		1.88
Total		43.04

*Found only in the acrocentric chromosome via in silico analysis.

**Figure 1:**
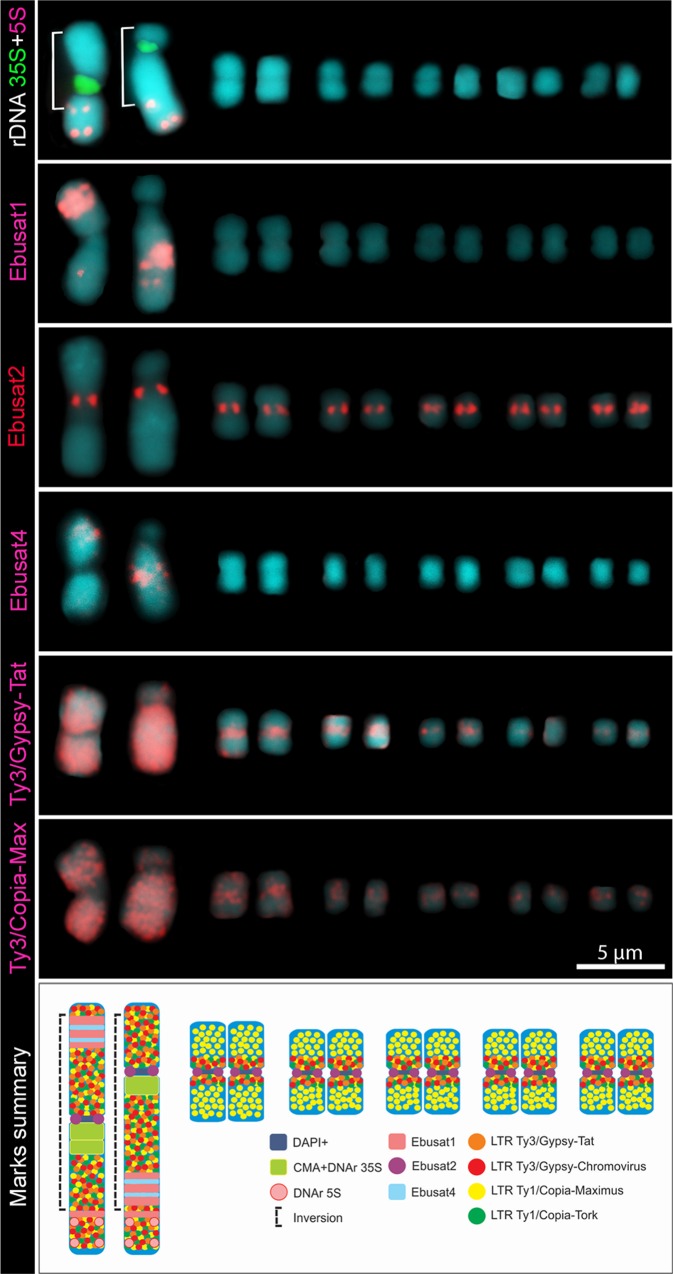
Comparative karyograms showing the distribution of repetitive elements in the *E. bulbosa* chromosomes. The brackets indicate the inverted chromosome region. The scheme below summarizes the repeat distribution.

The most abundant tandem repeat Ebusat1 localized at the large chromosome pair, mostly forming two interstitial blocks within the chromosome inversion: in the long arm of the acrocentric and in the short arm of the metacentric chromosome. A third weaker signal was observed outside of the inversion, in a distal position on the long arm of both chromosomes (**Figure 1**). Ebusat2 was located in the centromeric region of all chromosomes, showing similar hybridization intensities among all chromosomes (**Figure 1**). Both satDNAs colocalized with DAPI^+^ bands, except for the third smaller band of Ebusat1. For Ebusat3, no hybridization signals were detected, although the tandem repeat nature was confirmed by Dotter, and the amplified PCR fragments showed a ladder-like pattern.

All four most abundant LTR-retrotransposon lineages showed a high accumulation on the large chromosome pair, with dispersed labeling along the entire chromosome, except for the proximal region in chromosome I corresponding to the 35S ribosomal DNA (**Figures 1** and **S1**). Three of them (Tat, Chromovirus, and Tork) showed pericentromeric labeling on the small chromosomes, while Maximus showed a more scattered distribution, enriched proximally on small chromosomes (**Figures 1** and **S1**). These results demonstrate that both large and small chromosome types share the same repetitive DNA sequences, except for Ebusat1, which is present only in the large pair. However, except for Ebusat2, each chromosome type displays a specific chromosomal distribution for these repetitive sequences, with a higher abundance of repeats in the large chromosome pair.

In order to compare the DNA composition of the homologs of the large chromosome pair, microdissection was performed, and the isolated chromosome-specific DNA was sequenced. About 2,160 Mbp of the sequence reads from each homolog (within and outside the inversion) were used for comparative *in silico* analyses, representing ∼8× coverage for each chromosome. The reads were grouped into 13,279 clusters containing from 2 to 80,108 reads. The major 267 clusters, with a minimum of 0.01% of the chromosome DNA proportion, represented ∼27% of the chromosome DNA. Further, two different clustering analyses were run using two different sets of sequencing data each, showing similar results. The analysis revealed four satDNA families, Ebusat1, Ebusat2, and Ebusat3, as well as one previously undetected one, Ebusat4, seven LTR-RT lineages previously characterized in the genomic analysis, LINEs, DNA transposons, and ribosomal DNA sequences. Except for Ebusat2, the amount of all satDNAs seems to be higher in the larger chromosomes, in agreement with the distribution of Ebusat1 in this chromosome pair. In contrast, the LTR-RT lineages, except TAR in the metacentric chromosome, showed a similar or lower proportion within chromosome I than the total genome proportions. This may indicate a preferential amplification of the most abundant tandem repeats during the process of whole-genome amplification after microdissection (**Table S2**). Ebusat4 was mostly localized within the chromosome inversion, associated with the interstitial bands of the large chromosome pair as Ebusat1, in the long arm of the acrocentric and in the short arm of the metacentric chromosome (**Figure 1**). The hybridization signals were similar in both chromosomes of the large pair in disagreement with the differential proportion of Ebusat4 observed after analyzing the DNA composition of microdissected chromosomes. This difference could be explained by the uneven amplification of microdissected DNA (**Table S2**).

These results confirmed the presence of the major repetitive families in the large chromosome pair, as well as the presence of one new satDNA, Ebusat4, clearly enriched in both homologs of this pair. This distribution of repeats was not influenced by the occurrence of the nonrecombining inverted region of the large chromosome pair.

### The Chromatin Composition Differs Between Large and Small Chromosomes

As the identified repetitive DNA families showed a distinct chromosome type–specific distribution, we investigated the distribution of a subset of posttranslational histone modifications to understand whether the observed DNA composition differences are associated with a different chromatin organization.

**Figure 2:**
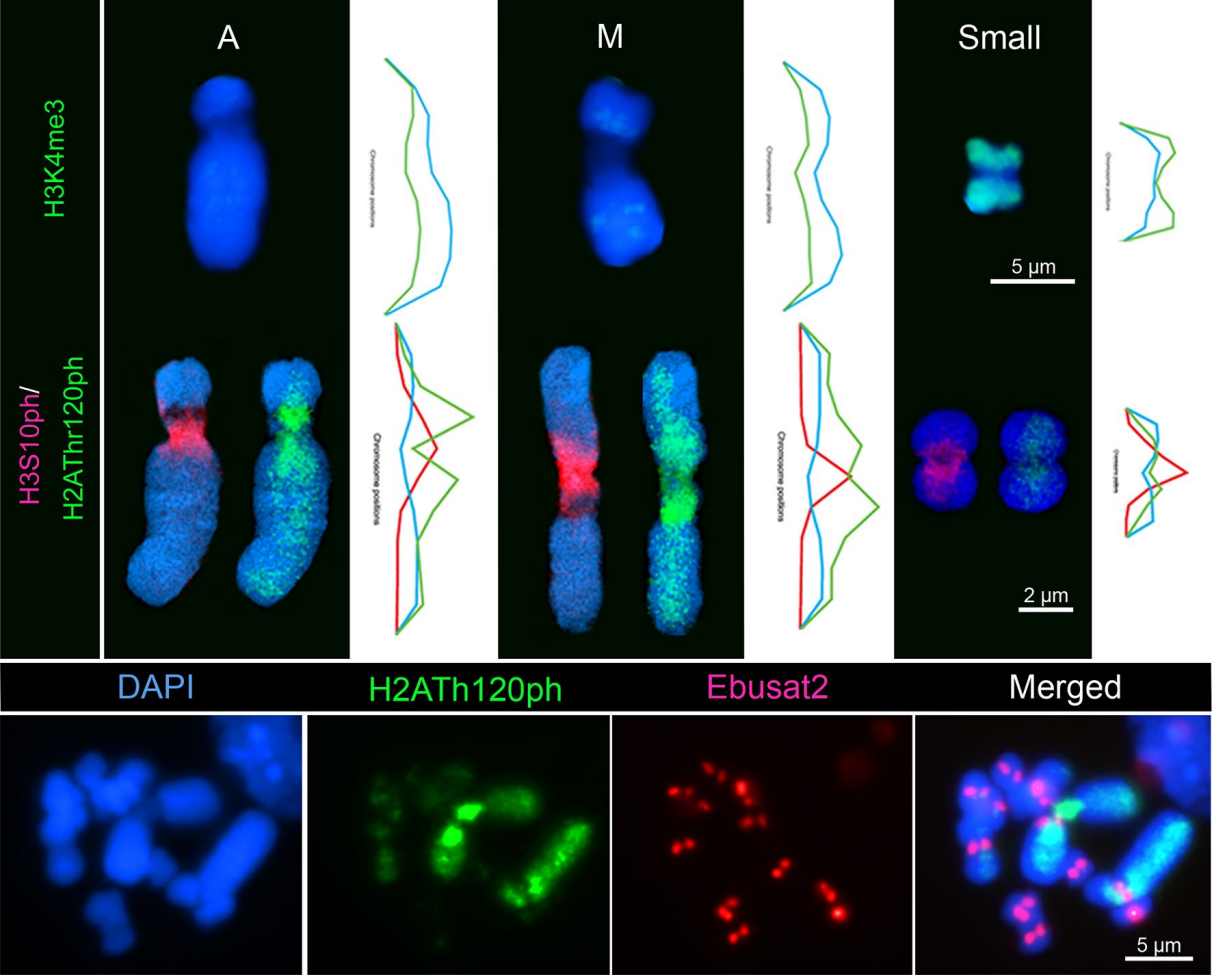
Chromatin organization differs between the large and small chromosomes of *E. bulbosa*. Top: Distribution of the euchromatin mark H3K4me3 on the large acrocentric (A), metacentric (M), and a small chromosome, each with a fluorescence intensity histogram of whole chromatin (DAPI) and H3K4me3 staining. Middle: Distribution of the pericentromeric histone modification marks H3S10ph and H2AThr120ph on the three chromosome types, with their respective fluorescence intensity histograms. Bottom: H2AThr120ph distribution along the chromosome arms and the Ebusat2 satDNA localization at the centromeres.

The small chromosomes of *E. bulbosa* showed strong labeling along the chromosome arms by the euchromatin histone mark H3K4me3, with weaker labeling in the pericentromeric regions. In contrast, both large homologs showed weaker labeling along the entire chromosomes (**Figure 2**). Histone modification marks for pericentromeric regions displayed contrasting results. While all chromosomes showed a pericentromeric distribution for phosphorylated H3 at serine 10, the large chromosome pair was strongly phosphorylated with H2AThr120ph at the proximal chromosome regions, flanking the pericentromeres and including the rDNA site. Toward distal arm regions, the phosphorylation of H2AThr120 gradually decreased. All small chromosomes showed a weak phosphorylation at the pericentromeres and weaker signals on the proximal and distal regions (**Figure 2** and **Movies S1**, **S2**). The different distribution of both histone modifications and between the small and large chromosomes was confirmed by fluorescence intensity measurements. Subsequent FISH with the centromeric Ebusat2 satellite repeats confirmed its localization between both proximal H2AThr120ph positive regions (**Figure 2**).

**Figure 3:**
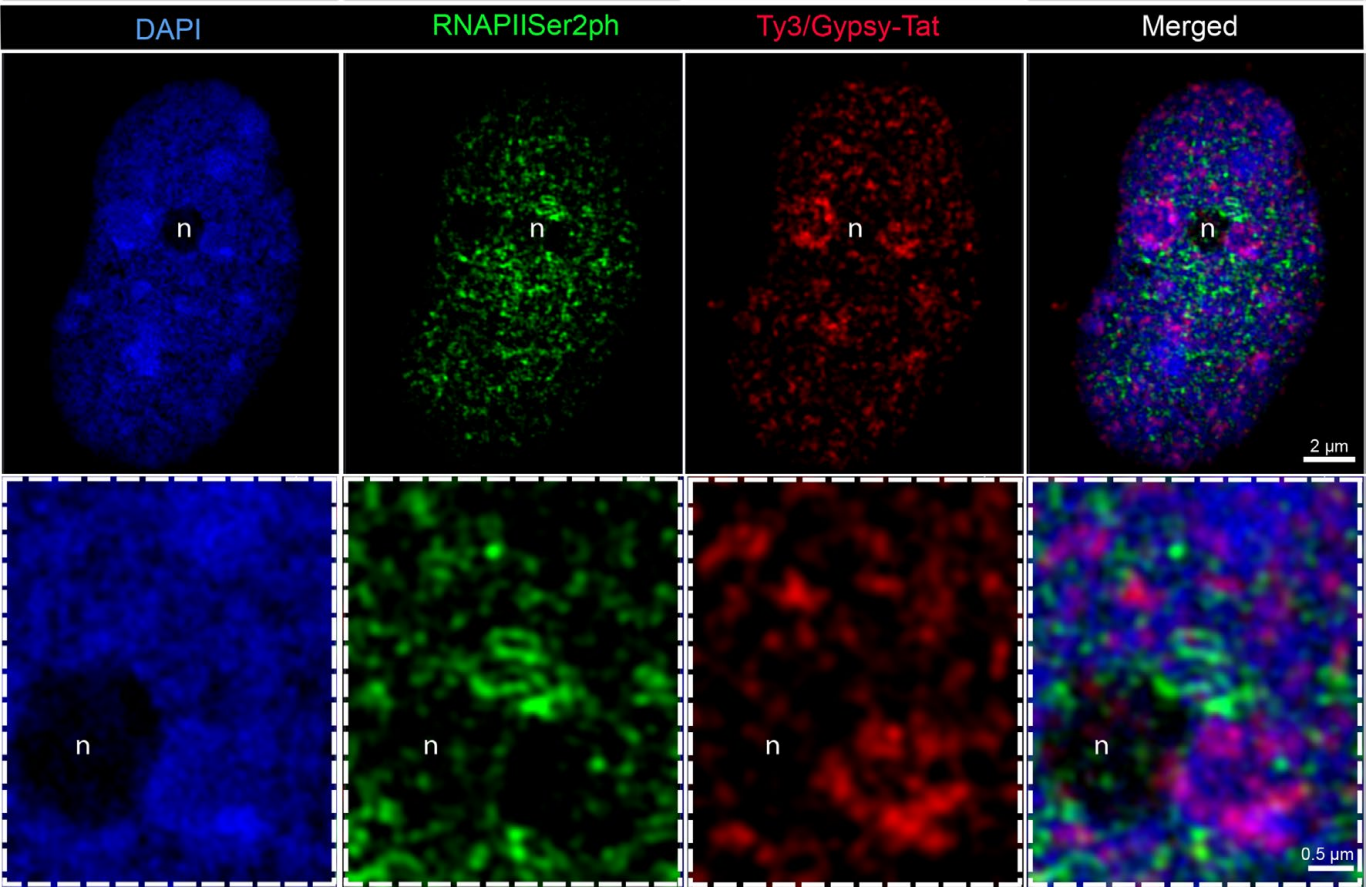
Distribution of RNAPIISer2ph and Ty3/Gypsy-Tat repetitive elements in *E. bulbosa* interphase nuclei, analyzed by super-resolution microscopy (SIM). Active RNAPII phosphorylated at serine 2 (RNAPIISer2ph) accumulates especially at LTR Ty3/Gypsy-Tat elements localized around the nucleolus (n).The enlarged region below is indicated by dashed rectangle.

We also analyzed whether different transcriptional activities between both chromosome types exist. Therefore, we applied specific antibodies against RNA polymerase II phosphorylated at serine 2 (RNAPIISer2ph) as a mark for transcriptional activity. Antibodies specific for the phosphorylation state of a peptide allow the discrimination between active and inactive RNAPII (Bourdon et al., 2012). For the elongation step of transcription, phosphorylation at serine 2 is required (Ni et al., 2004). RNAPIISer2 displayed a dispersed distribution in interphase nuclei, with a certain accumulation within the nucleus interior (**Figure 3**). Subsequent FISH with LTR-RT Ty3/Gypsy-Tat showed that the regions enriched with these repetitive elements, which are more abundant in the large chromosome pair and mostly present at the periphery of interphase nuclei, were only seldom associated with active RNAPII. In contrast, regions free of these repetitive elements, mostly in the central nuclear region, were enriched with RNAPIISer2ph (**Figure 3**).

Altogether, these results demonstrate that chromatin composition and transcriptional activity differ between large and small chromosomes. These differences are possibly a consequence of the different repetitive sequence composition in both chromosome types.

### Replication Dynamics Differ Between Large and Small Chromosomes

**Figure 4:**
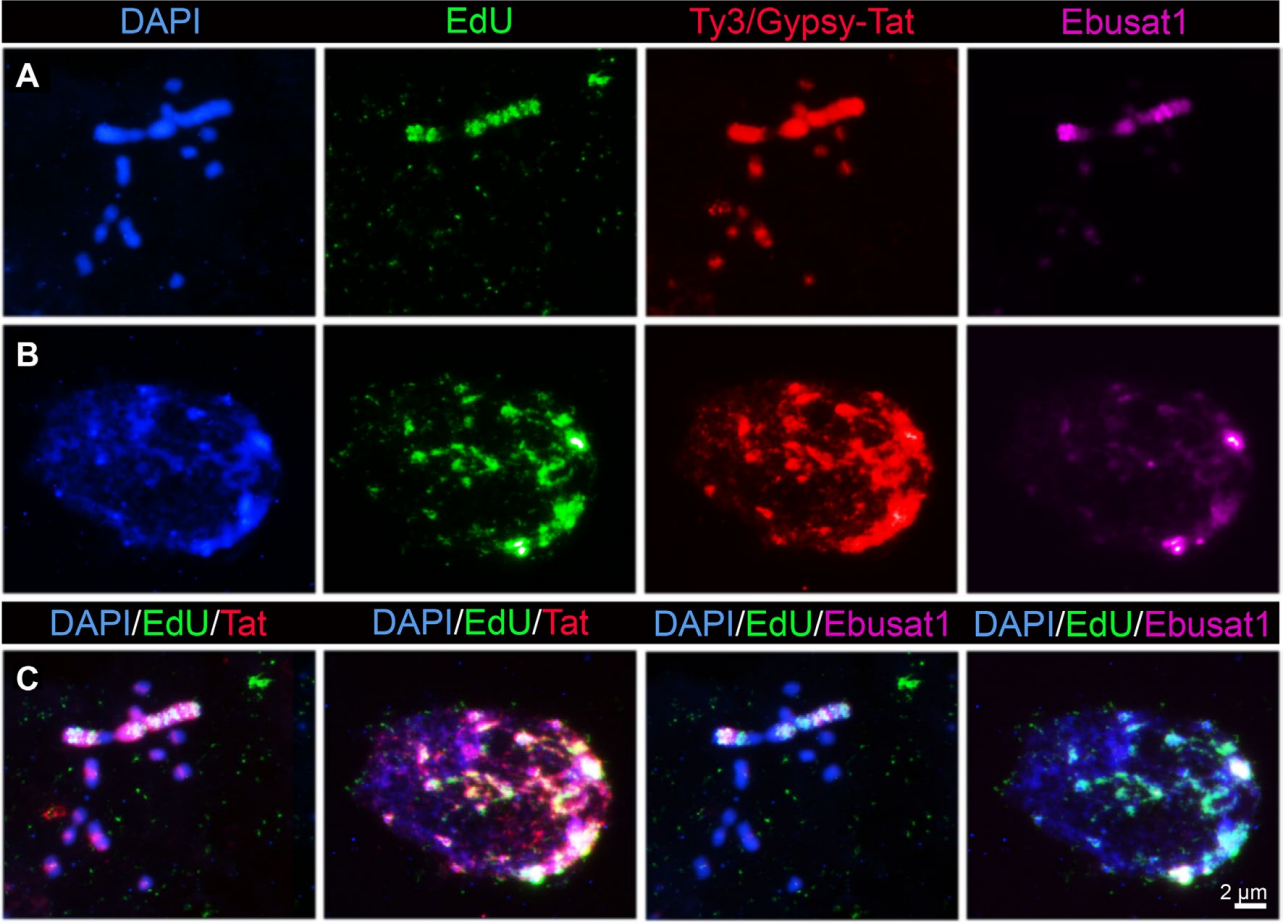
Replication dynamics differs between the large and small chromosomes of *E*.*bulbosa*. Metaphase chromosomes **(A)** and interphase nuclei **(B)** after EdU incorporation followed by LTR Ty3/Gypsy-Tat and Ebusat1 DNA localization. Colocalization is shown in **(C)**.

As large and small chromosomes present a different repeat and chromatin composition, replication analysis was performed to uncover a potentially different replication behavior of both chromosome types. Compared to the small chromosomes, the large chromosome pair incorporated EdU at a different time. Furthermore, incorporation into the large chromosomes was observed as bands along the chromosome arms (**Figure 4A**). Some cells showed this banding pattern together with the incorporation at the pericentromeric regions on all small chromosomes. Subsequent FISH with Ebusat1 and the Ty3/Gypsy-Tat element showed a partial colocalization between the EdU incorporation and these repeats in metaphase chromosomes as well as in interphase nuclei, mostly at the nucleus periphery (**Figures 4B, C**). These data indicate that the different chromosome structures also influence the replication timing, likely as a consequence of the presence of distinct repetitive sequences and chromatin compositions in the large and small chromosomes.

### Chromosomal Distribution of Repeats Is Similar in *E. latifolia*

**Figure 5:**
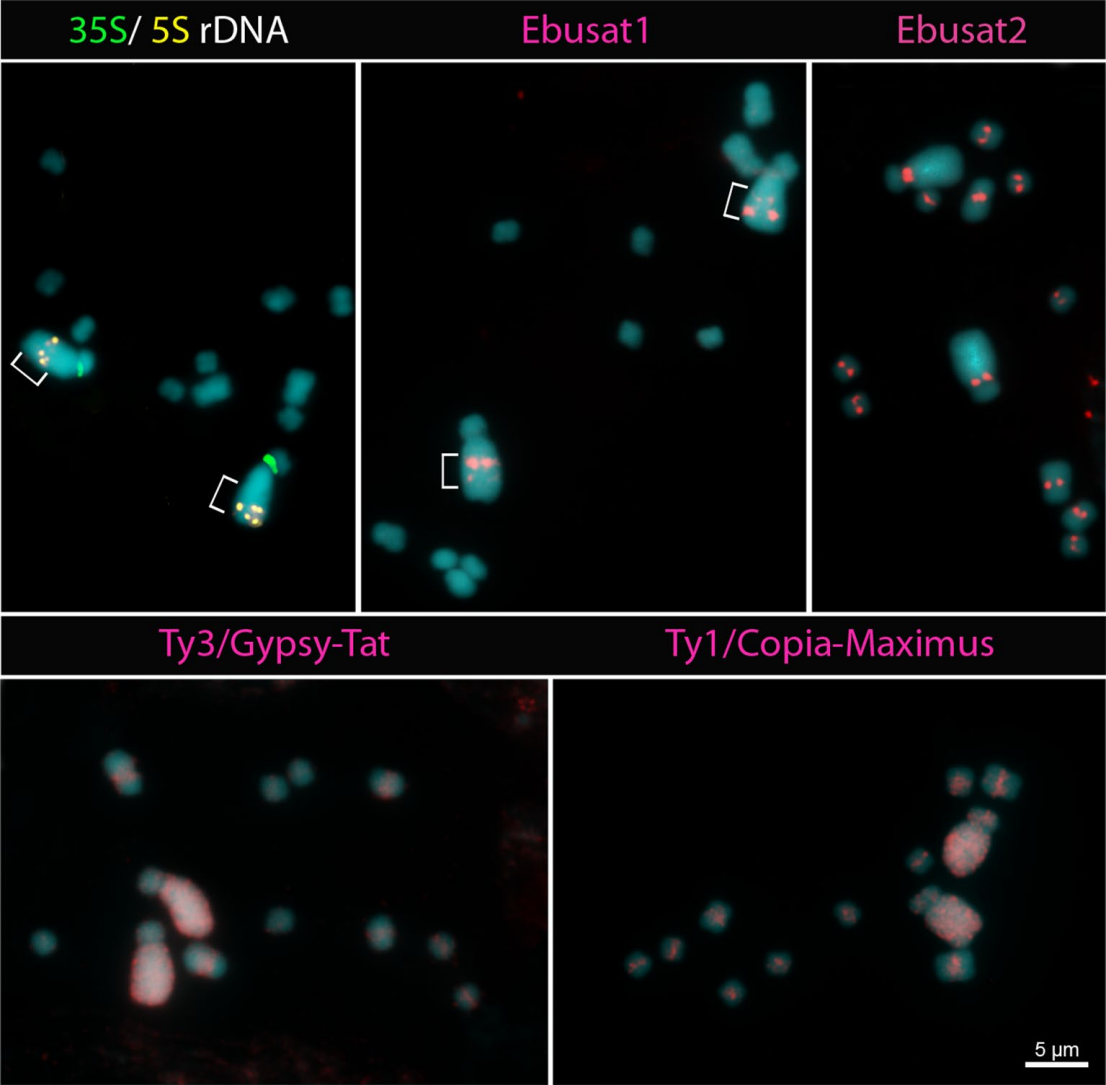
Distribution of repetitive elements in *E. latifolia*. The localization of the 5S, 35S ribosomal DNA (rDNA) sites and the Ebusat1 repeat indicates a paracentric inversion at the long arm of the large chromosome pair (brackets).

To analyze whether the subgenome repeat distribution is a specific feature of *E. bulbosa*, or a bimodal karyotype feature in the genus, we hybridized the major repeat sequences of *E. bulbosa* to the chromosomes of the sister species *E. latifolia* (2C = 1.4 pg). Both 35S and 5S rDNA sites were located on both of the large acrocentric homologs, with the 35S sites at the pericentromeric regions and the 5S sites at the interstitial regions of the long arms (**Figure 5**). The position of the 35S rDNA is similar to that in the acrocentric chromosome of *E. bulbosa*. The 5S rDNA sites are also duplicated in closer proximity as in *E. bulbosa*. However, in one of these homologs, both signals are located more distally, suggesting a paracentric inversion present in heterozygosity. The most abundant repetitive elements of *E. bulbosa*, both satDNAs (Ebusat1 and Ebusat2), the Ty3/Gypsy-Tat and Ty1/Copia-Maximus LTR-RT lineages, showed a similar distribution in *E. latifolia* as it was found in *E. bulbosa* chromosomes. Ebusat1 showed two distal bands at the long arms of the large acrocentric pair, one stronger and one weaker band, with inverted orientation between the homologs, confirming the paracentric inversion (brackets in **Figure 5**). Ebusat2 displayed a centromeric distribution in all chromosomes of the complement. Ty3/Gypsy-Tat and Ty1/Copia-Maximus LTR-RT exhibited an accumulation on the large acrocentric pair, similar as observed in *E. bulbosa*, irrespective of the position of the inversion. Ty1/Copia-Maximus was uniformly dispersed along the largest acrocentric chromosome pair. It was proximally distributed on all small chromosomes, but denser at the pericentromeric regions. Similarly, Ty3/Gypsy-Tat was uniformly dispersed along the large pair and showed a pericentromeric distribution in the small chromosomes (**Figure 5**). Chromosome signals from both satDNAs and LTR-RT were weaker in *E. latifolia* than in *E. bulbosa*. This suggests differences of these repeats families between both species, likely due to differences in sequence similarity and/or abundance. However, both species have the same distribution of repeat sequences, indicating that the chromosome set–specific repeat distributions are conserved in the genus and was not influenced by species-specific chromosome rearrangements. We suggest that this type of chromosome set–repeat distribution is a characteristic of bimodal karyotypes.

## Discussion

### Chromosomal Distribution of Repetitive DNA in *E. bulbosa* Reflects Its Bimodal Karyotype

Although repetitive sequences are enriched in the large chromosomes of several bimodal plant species (de la Herrán et al., 2001; Pedrosa et al., 2001), none of them showed a high degree of accumulation of LTR retroelements, together with satDNA, as seen in *Eleutherine*. The repetitive genome fraction of *E. bulbosa* is mainly composed of LTR retroelements, with more abundant Ty3/Gypsy-like than Ty1/Copia-like elements. Within eukaryote genomes, LTR-RT Ty3/Gypsy and Ty1/Copia are most abundant in plants (Kumar and Bennetzen, 1999), with the Ty3/Gypsy elements being the most abundant in the majority of angiosperm families (Weiss-Schneeweiss et al., 2015). All LTR retrotransposons were highly accumulated on the large chromosome pair of *Eleutherine*, showing a uniformly dispersed distribution, similar to the distribution observed for several mobile elements in large genomes (Lamb, 2006; Lamb et al., 2007). In contrast, the small chromosomes of *Eleutherine* accumulated the LTR retroelements only at the pericentromeric regions. This restricted distribution of repetitive sequences is typical for small genome species, e.g. in *Arabidopsis* (Heslop-Harrison et al., 1997), *Phaseolus* (Padeken et al., 2015), and *Brachiaria* (Santos et al., 2015). Thus, the differential distribution of retroelements appears to be related to the bimodal condition and is not influenced by the nonrecombining chromosome inversions in these species. The large and the small chromosome sets of *Eleutherine* constitute two distinct subgenomes with respect to the retroelement distribution.

The chromatin organization is partly similar in *Eleutherine* and bird species. The microchromosomes of birds are enriched in genes and hyperacetylated at histone H4K5, while macrochromosomes are gene-poor and hypoacetylated at histone H4K5 (McQueen et al., 1998). However, unlike the large chromosomes of *E. bulbosa*, bird macrochromosomes are poor in heterochromatin, with less 5-methylcytosine-rich regions than microchromosomes, probably related to CpG islands associated with the high gene content of the microchromosomes (Schmid and Steinlein, 2017). Nevertheless, some transposable elements show the differential chromosomal distribution in bimodal karyotypes of different animal groups. In birds, CR1-like retroelements are spread over nearly all chromosomes but have a higher density on macrochromosomes with a particular banding pattern (Coullin et al., 2005). The *Rex*6 transposable element is also densely distributed in the largest chromosome pairs in several species of *Podocnemis* turtles. It was suggested that *Rex*6 may influence the genomic structure, interfering with gene regulation (Noronha et al., 2016).

Retrotransposons are abundant components of large chromosomes in some plants with bimodal karyotypes, but differentiation of chromosome sets is less evident. The large chromosomes of South American *Hypochaeris* species are enriched by Ty1-Copia LTR-RT along their entire lengths, while small chromosomes lacked those elements in most of the long arms. However, this pattern is not maintained in other species of the genus, mainly not in Old World species (Morocco and Croatia; Ruas et al., 2008). On the other hand, in three species of the genus *Alstroemeria* with asymmetric chromosomes, the distribution of a Ty1-Copia like LTR-RT was equally dispersed over all chromosomes (Kuipers et al., 1998).

Here, two major satellite DNAs represent a large proportion of the genome (∼7%). Such a high proportion of one or two satDNA families is unusual in most plant species. Usually, many satDNA families with a low abundance or few satDNA families with slightly larger genome abundance were observed (Hemleben et al., 2007; Steflova et al., 2013). Contrary to what was suggested (Melters et al., 2013), the most abundant satDNA from *E. bulbosa* is not centromeric but accumulated interstitially exclusively in the large chromosome pair. The second most abundant satDNA is present in all centromeric regions. However, whether this location is associated with centromere functions has not yet been clarified. As in *Eleutherine*, large chromosomes of bimodal karyotypes in some plants accumulate satDNA. In *Muscari* species, a large chromosome specific satDNA found in *M. comosum* is conserved within the genus and has been proposed to mediate the increase of karyotype asymmetry (de la Herrán et al., 2001). One of the major components of intercalary heterochromatin on the large chromosomes of *O. longibracteatum* was also a specific satDNA (Pedrosa et al., 2001). Therefore, as clearly observed in *Eleutherine*, large and small chromosomes within a bimodal karyotype can maintain a differential DNA composition. This can cause structural and functional differences between these subgenomes.

### Large and Small Chromosomes Differ in Heterochromatin, Gene Content, and Replication Timing

Chromatin-associated H3K4me3 and H2AThr120ph histone marks differentiate the large and small chromosomes in *E. bulbosa*. The dense distribution of H3K4me3 along all small chromosomes is associated with euchromatin, as it was observed also for other histone modification marks indicating euchromatin in the small chromosomes of this and other species with small genomes (Feitoza and Guerra, 2011). The less intense distribution of H3K4me3 on the large chromosomes is likely associated with a higher proportion of heterochromatin, as revealed by the repeat distribution of in the present work, as well as by 5-methylcytosine and H3K9me2 localization (Feitoza and Guerra, 2011).

The so-called universal histone modification mark for the pericentromeric region, H2AThr120ph (Dong and Han, 2012, Demidov et al., 2014), did not exclusively label the pericentromeric region in *E*.*bulbosa* as defined by the H3S10ph mark. The large chromosomes also showed intense H2ATh120ph signal along their lengths. This suggests that the proximal chromatin composition may differ between both chromosomes sets and that it is rather related to the chromosome structure than to centromere function. A comparable atypical distribution of this histone mark was found for the large Y chromosome of *Coccinia grandis* (Sousa et al., 2016).

Large and small subgenomes of *Eleutherine* also differ in their replication timing. Large chromosomes showed a banding pattern after EdU incorporation. This suggests that, within these chromosomes, there are regions that perform replication at a different time than those of the small chromosomes. This could be due to the high repeat composition within large chromosomes, as confirmed by the colocalization of some repetitive elements with EdU bands. Differential replication timing was also reported for chicken macrochromosomes displaying late replication and microchromosomes showing early replication, both related to their different gene content (McQueen et al., 1998). This assumption could also be valid for *E. bulbosa*, because the distribution of active RNAPII in interphase nuclei suggests chromosome regions with different transcription activities. Chromatin enriched in active RNAPII did not colocalize to repeat-rich nuclei regions and is possibly associated with small chromosomes. In addition, a weaker RNAPIISer2ph labeling and more repetitive DNA were observed at the nuclear periphery, possibly associated with large chromosomes. Together, these data suggest that the large chromosomes of *Eleutherine* are mainly composed of heterochromatin and heterochromatin-like, early-condensing euchromatin (Guerra, 1988; Feitoza and Guerra, 2011). They have a lower gene density and partially replicate later. In contrast, small chromosomes are composed mainly of euchromatin, are gene-rich, and replicate earlier. Thus, the large and small chromosomes represent structurally and functionally differentiated subgenomes within the same species.

### The Bimodal Chromosomal Organization Is Maintained Within the Sister Species *E. latifolia*

Both investigated *Eleutherine* species display a similar repeat distribution, indicating that these repeats originated and underwent a chromosome-type specific accumulation before the separation of these two species. The newly discovered chromosome inversion in *E. latifolia*, a paracentric inversion in the long arm of one homolog of the large chromosome pair, also involved Ebusat1. One breakpoint is apparently close to the breakpoint of the *E. bulbosa* pericentric inversion. Both events may suggest that this chromosome pair is prone to chromosome rearrangements, possibly due to its highly repetitive sequence content. Repetitive sequences are potential sites for chromosome rearrangements through homology-directed recombination repair using ectopic homologous repeats as a template (Charlesworth et al., 2005). Although vegetative reproduction in *Eleutherine* could be responsible for the maintenance of both inversions in heterozygous condition, the recurrent rearrangements in this chromosome pair are possibly associated with a strong purifying selection, with lethality under homozygous conditions. This may lead to permanent heterozygosity of this chromosome pair, even in individuals propagated *via* seeds. First, we hypothesized that the chromosomal inversion in *E. bulbosa* could have led to repetitive sequence accumulation on the large chromosome pair. However, since the repeat distribution is uniform along the entire large chromosome pair, even outside the inverted region, and is similar in both *Eleutherine* species, it is more likely that it is related to the bimodal structuration and function of these karyotypes, rather than the consequence of chromosome inversions.

### How could a Bimodal Karyotype Evolve in *Eleutherine*?

Different types of bimodal karyotypes exist, since not in all bimodal species different chromatin and sequence composition between large and small chromosomes exist, as presented for *Eleutherine*. There are different hypotheses for the origin of bimodal karyotypes. Interspecific hybridization appears not to apply to *Eleutherine*, since the bimodal karyotype is characteristic for the whole tribe Tigridieae, with no evidence of allopolyploidy for its origin (Moraes et al., 2015).

**Figure 6:**
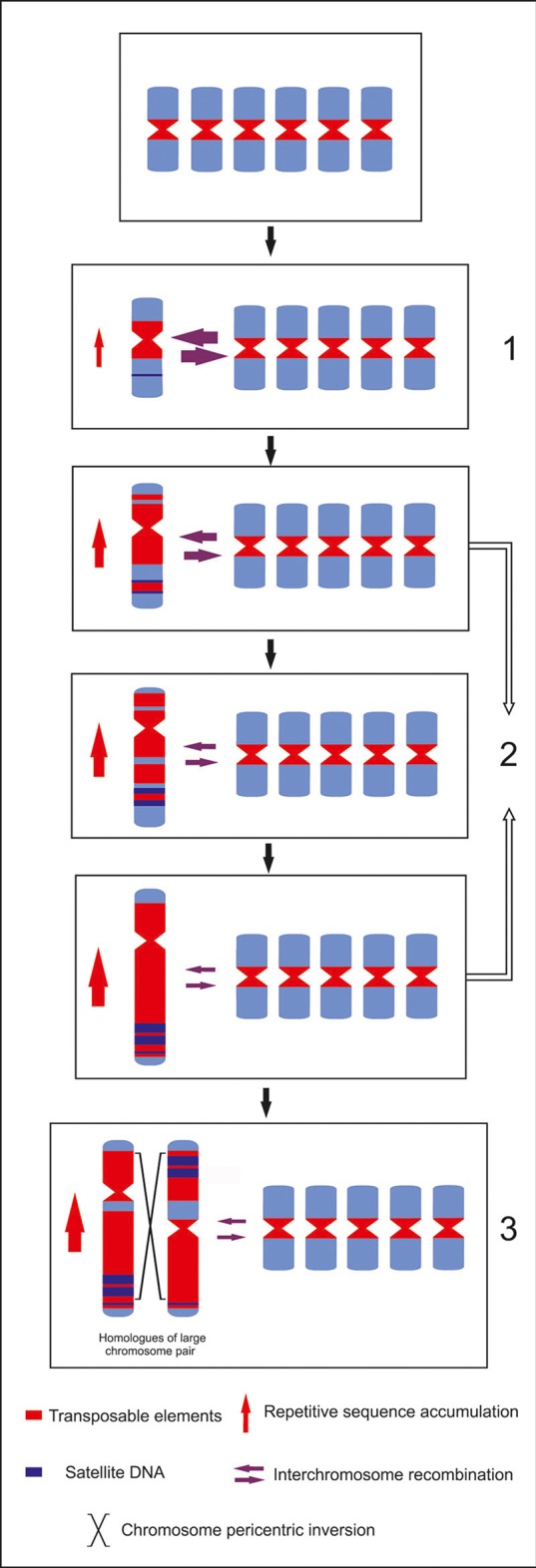
Bimodal karyotype evolution model for *E. bulbosa*. From a symmetric to a bimodal karyotype: (1) random increase of retroelements and satellite DNA; (2) preferential maintenance of retroelements accumulating in the large-chromosome subgenome with increasing chromosome size and decreasing gene density; (3) pericentric inversions lead to the heteromorphic homologs on the large chromosome pair, maintained by recessive lethality with selection advantages in the heterozygote status.

Although paleo-alloploidization or ancestral chromosomal rearrangements cannot be completely ruled out, our data indicate an increase of the size of one set of chromosomes due to a differential repetitive sequences accumulation. This phenomenon was suggested for species of the *Muscari* genus. Differential accumulation of one satDNA was associated to the increase in size of a subset of chromosomes (de la Herrán et al., 2001). It is possible that an initial random accumulation of repetitive sequences in one chromosome pair in *Eleutherine* ancestral gradually increased its size and led to its chromatin differentiation, differential replication, and transcription behavior. Consequently, this may lead to a higher repetitive sequence accumulation and divergence of different repetitive families in the large chromosomes, which is less deleterious than in the more gene-rich small chromosomes (Vosa, 2005; see **Figure 6**). In both *Eleutherine* species, this repetitive environment provided a special background for chromosomes inversion.

In chicken, the microchromosomes present an increased rate of meiotic recombination compared to the macrochromosomes, perhaps due to a gene composition that favors meiotic recombination. This could lead to evolutionary pressure for an increase of gene density on small chromosomes (Smith et al., 2000; Rodionov et al., 2002). The negative feedback between repeats and recombination may be intensified by intrachromosomal rearrangements, also contributing to suppress recombination and subgenome differentiation in bimodal karyotypes, as our results suggested for *Eleutherine*. The possible gradual increase of meiotic recombination within the small chromosome set and, in consequence, a decrease of recombination between small and large chromosome sets could also favor the differentiation of the structure and function of both chromosome set and the maintenance of the bimodal karyotypes over time.

## Data Availability Statement

The raw sequencing data is available in Genbank Bioproject PRJNA549830. The sequences of the repeats found in this work is available in Genbank database as MK228130-MK228135.

## Author Contributions

MB, AH, and AP-H designed the study. MB and MV carried out the bioinformatics studies. SD carried out the chromosome microdissection with the help of MB. VS carried out the super-resolution microscopy. MB carried out the cytogenetic, molecular and flow cytometry experiments, interpreted the results, and wrote the manuscript with the help of AH and AP-H. All authors read and approved the final manuscript.

## Funding

We thank the Fundação de Amparo à Ciência e Tecnologia do Estado de Pernambuco (FACEPE), the Conselho Nacional de Desenvolvimento Científico e Tecnológico (CNPq Nº 310804/2017-5), and the Leibniz Institute of Plant Genetics and Crop Plant Research (IPK), Germany, for financial support. This study was supported in part by the Coordenacão de Aperfeicoamento de Pessoal de Nıvel Superior–Brasil (CAPES, Finance Code 001) and MB received a scholarship CAPES Nº 99999.003674/2015-00.

## Conflict of Interest

The authors declare that the research was conducted in the absence of any commercial or financial relationships that could be construed as a potential conflict of interest.

The handling editor and reviewer HW-S declared their involvement as co-editors in the Research Topic, and confirm the absence of any other collaboration.

## Supplementary Material

Video S1 Movie showing the differential chromatin organization within the centromeric region of E. bulbosa chromosomes mitotic complement. Pericentromeric histone modification marks H3S10ph (red) and H2ATh120ph (green), on both chromosome types. Chromosomes are stained with DAPI (blue).

Video S2 Movie showing the differential chromatin organization within the centromeric region of E. bulbosa metacentric chromosome. Pericentromeric histone modification marks H3S10ph (red) and H2ATh120ph (green). Chromosomes are stained with DAPI (blue).
