# Supplementary material for: Together But Different: The Subgenomes of the Bimodal Eleutherine Karyotypes Are Differentially Organized
Source: Front Plant Sci. 2019 Oct 7;10:1170. doi: 10.3389/fpls.2019.01170 (PMC6791338; doi:10.3389/fpls.2019.01170)
Supplement: Supplementary file 3 [file Image_1.pdf]

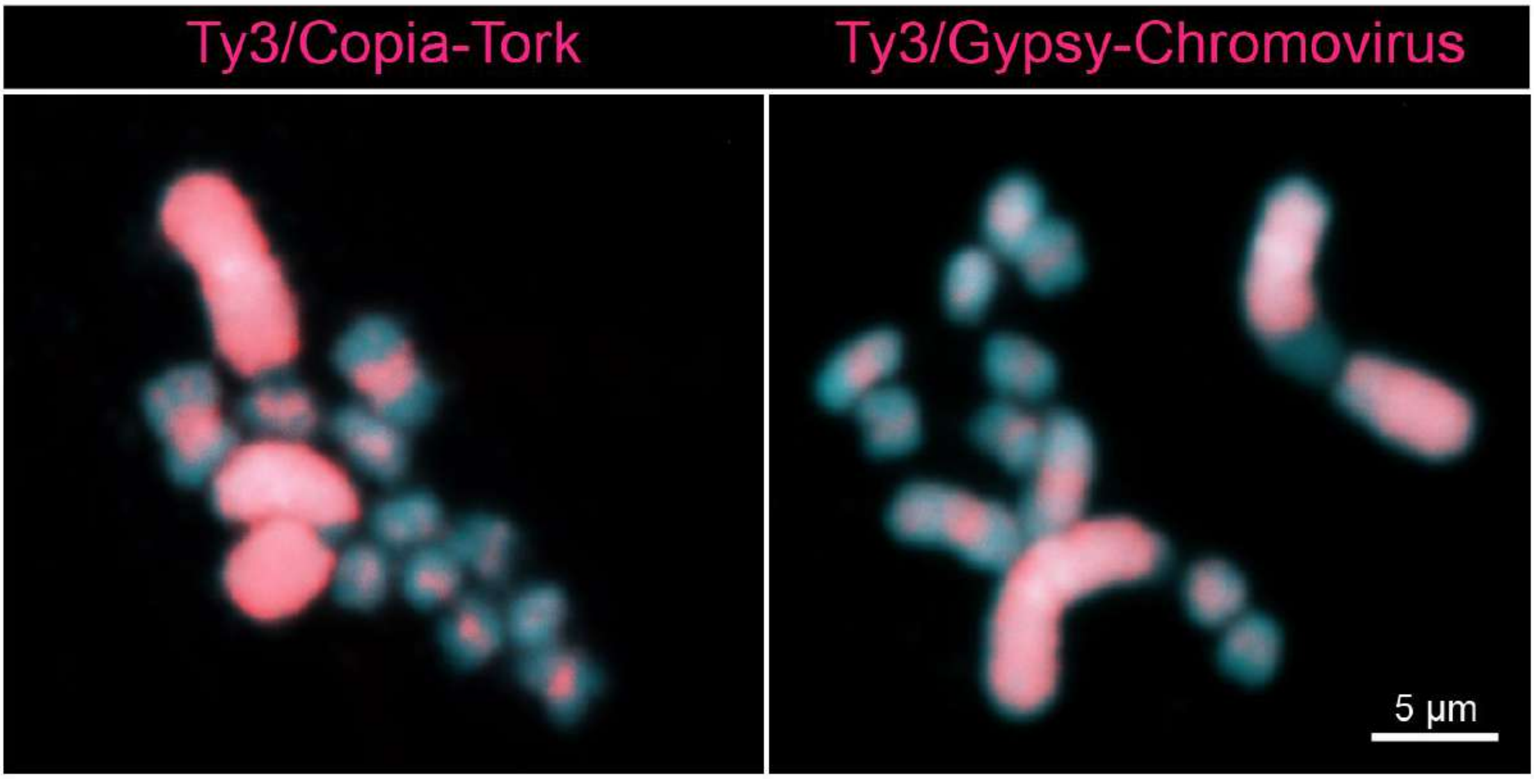

Figure S1. Distribution of LTR-retrotransposons Ty1/Copia-Tork and Ty3/Gypsy-Chromovirus elements in E. bulbosa. Both types of repeats are highly enriched along the large chromosome pair and the at the pericentromeric region in all small chromosomes.
