# Supplementary material for: Together But Different: The Subgenomes of the Bimodal Eleutherine Karyotypes Are Differentially Organized
Source: Front Plant Sci. 2019 Oct 7;10:1170. doi: 10.3389/fpls.2019.01170 (PMC6791338; doi:10.3389/fpls.2019.01170)
Supplement: Supplementary file 4 [file Table_1.docx]

Table S1 List of primers used to amplify *E. bulbosa* repetitive elements.

| Repetitive element | Primer name | Primer sequence |
| --- | --- | --- |
| Ebusat1 | Ebusat1F | TTGCCAATTGACATATTGCCAT |
| Ebusat1 | Ebusat1R | TCAATATGTCAATTGGCAATATGTC |
| Ebusat2 | Ebusat2F | TTTAGAACTTTGGTAAACTCTGCG |
| Ebusat2 | Ebusat2R | AGTTTTACGTAGTTTCAAACAACCT |
| Ebusat3* | Ebusat3F | CCGATTTGAGTATGTGCTTGAAAAA |
| Ebusat3* | Ebusat3R | GCAAATTCGCATACTATAACTCACAG |
| Ebusat4 | Ebusat4-F | AACAAACACAAACATGCACACA |
| Ebusat4 | Ebusat4-R | TGCATCTCCGTATGTATGGCT |
| LTR Ty3/Gypsy Chromovirus | CL126ctg87F | GGCACCGTACGAAGCCTTAT |
| LTR Ty3/Gypsy Chromovirus | CL126ctg87R | TTCCAAGTGGCTTCCTCGTG |
| LTR Ty1/Copia Maximus | CL19ctg162F | TTTATCATGGTACGGGCCGC |
| LTR Ty1/Copia Maximus | CL19ctg162R | GTGATGCTTGCAACTTGGGAA |
| LTR Ty3/Gypsy Tat | CL29ctg117F | GCCGATCGATCCACCTTCAT |
| LTR Ty3/Gypsy Tat | CL29ctg117R | TTTTCCTCCAGCATGGCGAT |
| LTR Ty1/Copia Tork | CL89ctg39F | ACATTGTGGCATCGTCGACT |
| LTR Ty1/Copia Tork | CL89ctg39R | CGTACGGTTCATCCTCTCCG |

*No signal at chromosomes.
