# Supplementary material for: Together But Different: The Subgenomes of the Bimodal Eleutherine Karyotypes Are Differentially Organized
Source: Front Plant Sci. 2019 Oct 7;10:1170. doi: 10.3389/fpls.2019.01170 (PMC6791338; doi:10.3389/fpls.2019.01170)
Supplement: Supplementary file 5 [file Table_2.docx]

Table S2 Repetitive element proportions (%) in the total genome of *E. bulbosa*. Estimated proportion of each repeat in the metacentric and acrocentric homologue of pair I, with a preferential vies amplification of the most abundant repetitive families during the whole genome amplification after microdissection.

| Repetitive element | | Total genome | Metacentric | Acrocentric |
| --- | --- | --- | --- | --- |
| Satellite | Ebusat 1 | 4.99 | 14.8 | 5.29 |
|  | Ebusat 2 | 2.16 | 0.94 | 1.28 |
|  | Ebusat 3 | 1.4 | 14.88 | 14.0 |
|  | Ebusat 4 | - | 0.02 | 6.22 |
| LTR-Ty3/Gypsy | Chromovirus | 1.76 | 0.30 | 1.71 |
|  | Tat | 14.26 | 0.07 | 0.06 |
| LTR-Ty1/Copia | Maximus | 1.78 | 0.30 | 0.68 |
|  | Tork | 1.46 | 0.88 | 0.01 |
|  | TAR | 1.23 | 2.48 | 0.35 |
|  | AleII | 1.19 | 0.25 | 0.07 |
|  | Angela | 0.98 | 0.18 | 0.002 |
|  | Ivana/Oryco | 0.31 | - | - |
|  | Bianca | 0.48 | - | - |
|  | AleI.Retrofit | 0.031 | - | - |
| Unclassified LTR |  | 6.39 | 9.75 | 21.9 |
| LINE |  | 0.19 | 0.63 | 0.45 |
| DNA Transposons |  | 2.08 | 2.64 | 5.36 |
| rDNA |  | 0.53 | 1.11 | - |
| Microsatellite |  | 0.33 | - | - |
| Unclassified |  | 1.88 | 21.5 | 1.3 |
| Total |  | 43.04 | 68.8 | 58.8 |
